# Mercury deposition in Western Tethys during the Carnian Pluvial Episode (Late Triassic)

**DOI:** 10.1038/s41598-021-96890-8

**Published:** 2021-08-30

**Authors:** Mina Mazaheri-Johari, Piero Gianolla, Tamsin A. Mather, Joost Frieling, Daoliang Chu, Jacopo Dal Corso

**Affiliations:** 1 Department of Physics and Earth Sciences, University of Ferrara, Ferrara, Italy; 2 Department of Earth Sciences, University of Oxford, South Parks Road, Oxford, UK; 3 State Key Laboratory of Biogeology and Environmental Geology, School of Earth Sciences, China University of Geosciences, Wuhan, China

**Keywords:** Palaeoclimate, Stratigraphy

## Abstract

The Late Triassic Carnian Pluvial Episode (CPE) was a time of biological turnover and environmental perturbations. Within the CPE interval, C-isotope and sedimentary records indicate multiple pulses of depleted carbon into the atmosphere–ocean system linked to discrete enhancements of the hydrological cycle. Data suggest a similar cascade of events to other extinctions, including being potentially driven by emplacement of a large igneous province (LIP). The age of the Wrangellia LIP overlaps that of the CPE, but a direct link between volcanism and the pulsed CPE remains elusive. We present sedimentary Hg concentrations from Western Tethys successions to investigate volcanic activity through the previously established CPE global negative C-isotope excursions (NCIEs). Higher Hg concentrations and Hg/TOC are recorded just before and during NCIEs and siliciclastic inputs. The depositional settings suggest volcanic Hg inputs into the basins over the NCIEs rather than increases of Hg drawdown or riverine transport. Differences in Hg and Hg/TOC signals between the basins might be linked to coeval LIP style or the temporal resolution of the sedimentary successions. Overall, our new data provide support for a link between pulses of Wrangellia LIP volcanism, NCIEs, and humid phases that mark the CPE in the Western Tethys.

## Introduction

**Figure 1:**
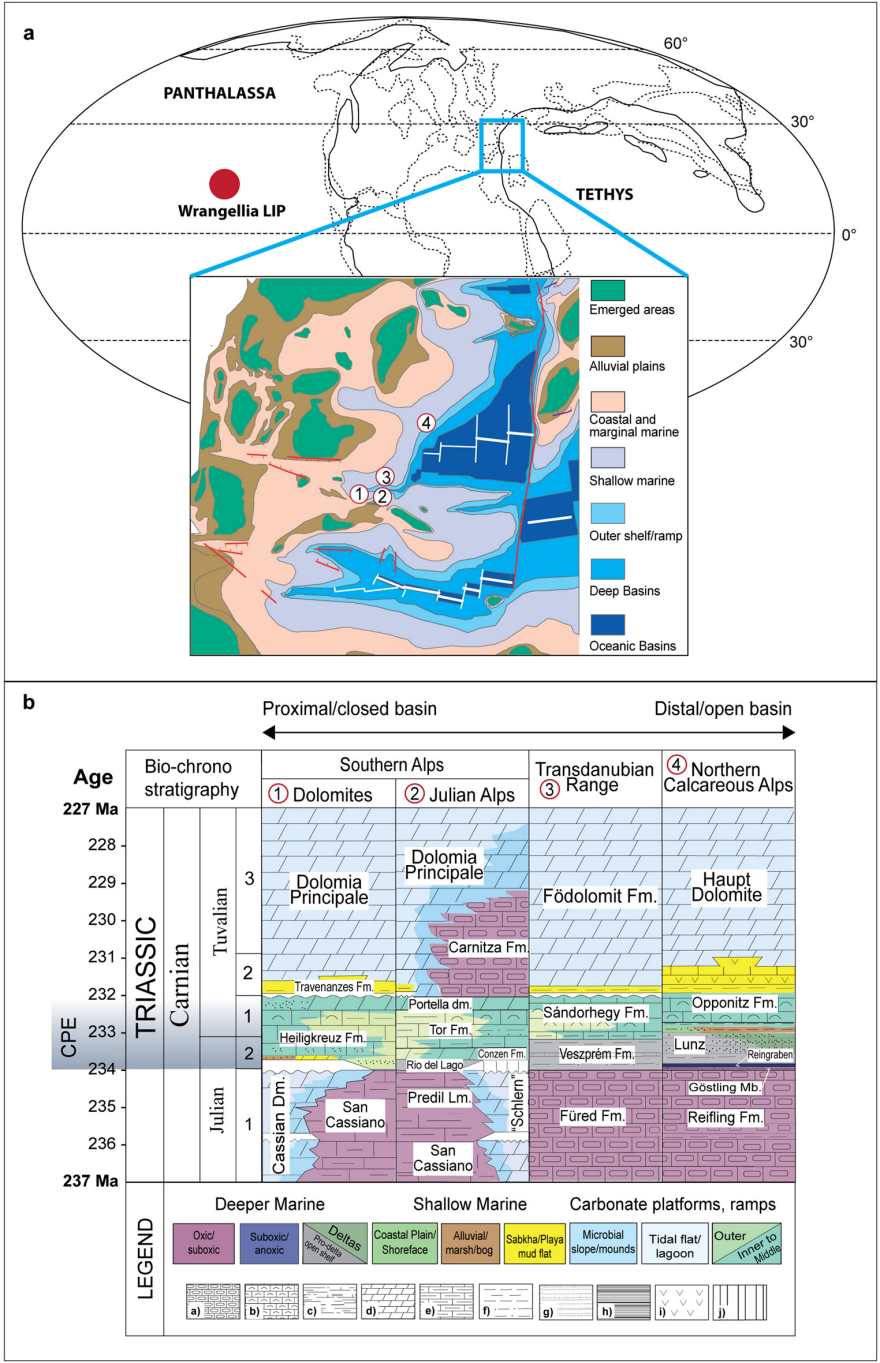
(**a**) Late Triassic palaeogeography and position of the studied successions in the Western Tethys (1 = Dolomites; 2 = Julian Alps; 3 = Transdanubian Range; 4 = Northern Calcareous Alps); the map is modified using QGIS (version 3.20.1, https://qgis.org/en) after ref.^14^, (**b**) Lithostratigraphic scheme for the Carnian formations in the studied areas. Julian 1 = *Trachyceras* zone, Julian 2 = *Austrotrachyceras austriacum* zone; Tuvalian 1 = *Tropites dilleri* zone , Tuvalian 2 = *Tropites subbullatus* zone , Tuvalian 3 = *Anatropites spinosus* zone. Lithology: a = Cherty limestone, b = Bioclastic limestone, c = Limy marlstone, d = Dolomite, e = Marly limestone, f = Siltstone, g = Sandstone, h = Black shale, i = Evaporites, j = Hiatus.

The Carnian Pluvial Episode (CPE, Late Triassic) was an interval of global warming and enhanced hydrological cycle coupled to extinctions and major radiations among terrestrial and marine taxa, giving rise to new modern-style ecosystems^1–11^. The CPE lasted for 1.2–1.6 Myrs, in the late Julian–early Tuvalian of the Carnian^12–14^ (Fig. 1), and was marked by repeated carbon-cycle perturbations, as evidenced by multiple negative carbon-isotope excursions (NCIEs) ^6–8,13,15–22^. The NCIEs indicate large injections of ^13^C-depleted carbon into the exogenic C-cycle reservoirs, each of which just precedes increases of continental runoff, as chiefly observed in the successions of the Western Tethys, and changes in carbonate sedimentation^7,8,21,23,24^. Understanding the triggers of the CPE is crucial given its important juncture in Earth history and the parallels with the cascade of events associated with other mass extinctions. Coeval volcanic activity has previously been invoked as a cause for the CPE’s NCIEs, both in terms of direct C emissions and as a trigger for positive C-cycle feedbacks (e.g., release of ^13^C-depleted C from ocean floor clathrates)^6,13,17^. The emplacement of the Wrangellia oceanic plateau, a submarine large igneous province (LIP), has been the main candidate for this scenario^6,8,9,15–18^. However, besides age overlap^25^ and Os isotope data from deep water successions of Panthalassa that show Wrangellia started in the Early Carnian *Trachyceras* ammonoid Zone, hereafter referred to as Julian 1^16^, further evidence for volcanic activity in the sedimentary record has been lacking. Hence, a direct temporal link between LIP volcanism and the environmental changes has been difficult to substantiate. Sedimentary Hg concentrations have been used to track volcanic activity during intervals of mass extinction and global environmental change^26–29^. Though other factors can at least partially control Hg deposition in terrestrial and marine settings^30,31^, increases of sedimentary Hg concentration at or directly below the level of mass extinction, C-cycle and/or environmental perturbations have been used as evidence for the influence of volcanism^28^. Here we analysed Hg and total organic carbon (TOC) concentrations in four Carnian marine sedimentary sequences of the Western Tethys to evaluate whether enhanced Hg loading and volcanic activity coincided with the C-cycle and hydrological perturbations across the CPE.

## Study area

We analyzed 243 mudstone and shale samples for Hg and TOC from 4 different areas of the Western Tethys realm: Southern Alps (Dolomites and Julian Alps) in Italy, Northern Calcareous Alps (Lunz) in Austria, and Transdanubian Range (Balaton) in Hungary (Fig. 1). The studied successions encompass the CPE and are well-calibrated with ammonoid, conodont, and sporomorph biostratigraphy^7,8,32^. In the Dolomites, 20 samples are from the Milieres section and 30 samples from the Heiligkreuz section, encompassing the Heiligkreuz Formation (*A. austriacum*–*T. dilleri* ammonoid zones = Julian 2–Tuvalian 1)^7,8^. In the Julian Alps, 58 samples were collected at Cave del Predil (Rio Conzen and Rio delle Cascate sections). The succession encompasses the Predil Limestone, the Rio del Lago Formation, the Conzen Formation, and part of the Tor Formation (*Trachyceras*–*A. austriacum* zones = Julian 1–2)^8,32^. In the Transdanubian Range, a total number of 73 samples were analyzed from two cores (BFÜ-1 and MET-1; Rostási et al., 2011). 39 samples are from BFÜ-1 and 34 samples from MET-1, covering the Füred Limestone and the Veszprém Marl Formation (*Trachyceras*–*A. austriacum*). In the Northern Calcareous Alps (Austria), we analyzed 62 samples from Steinbach and Polzberg sections^7,19^, encompassing the Reifling Formation, the Göstling Member and the Reingraben Formation (*Trachyceras*–*A. austriacum* zone = Julian 1–2). A detailed description of the studied successions is in the Supplementary Information.

## Results

**Figure 2:**
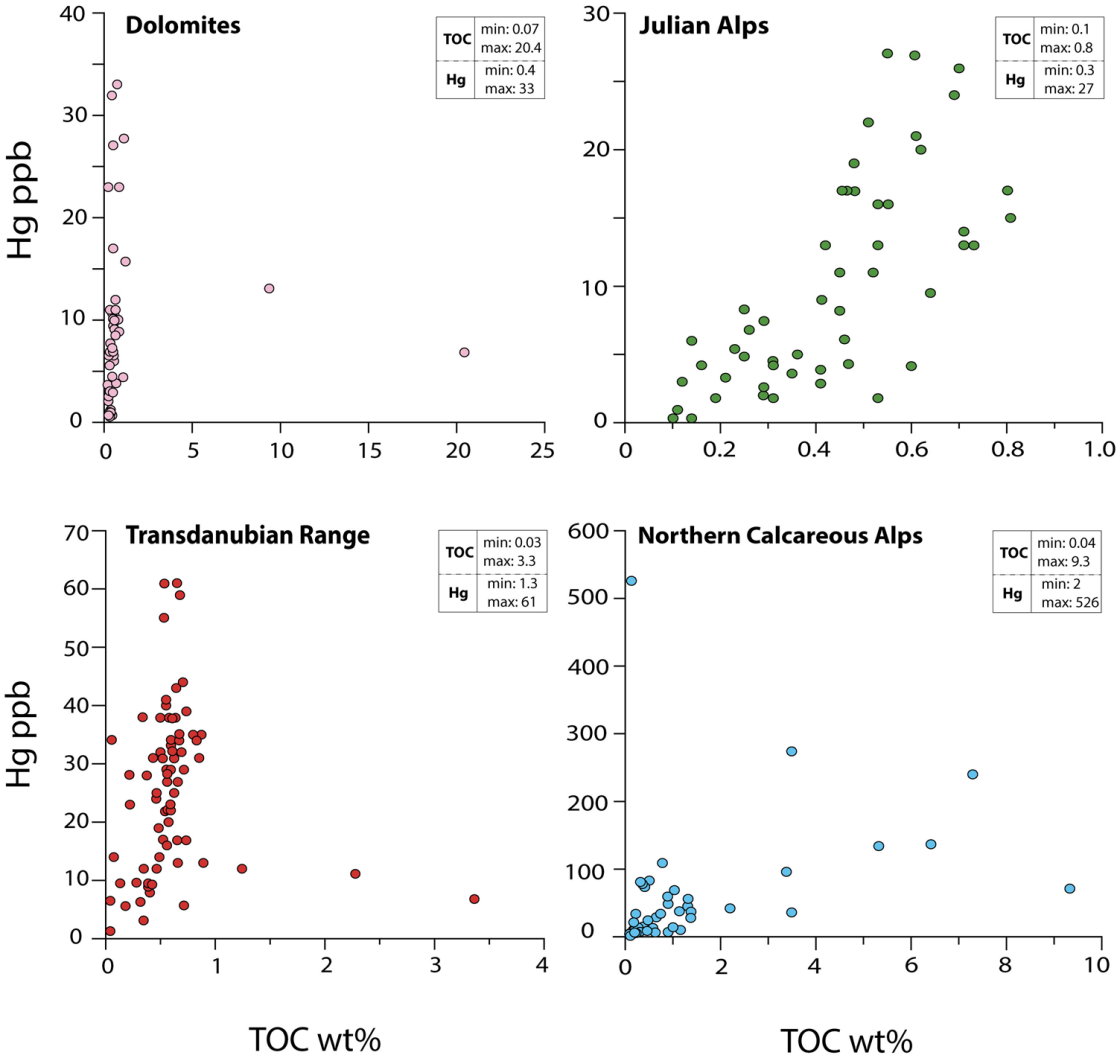
Hg *vs* TOC values from the studied successions of the Western Tethys (Fig. 1).

**Figure 3:**
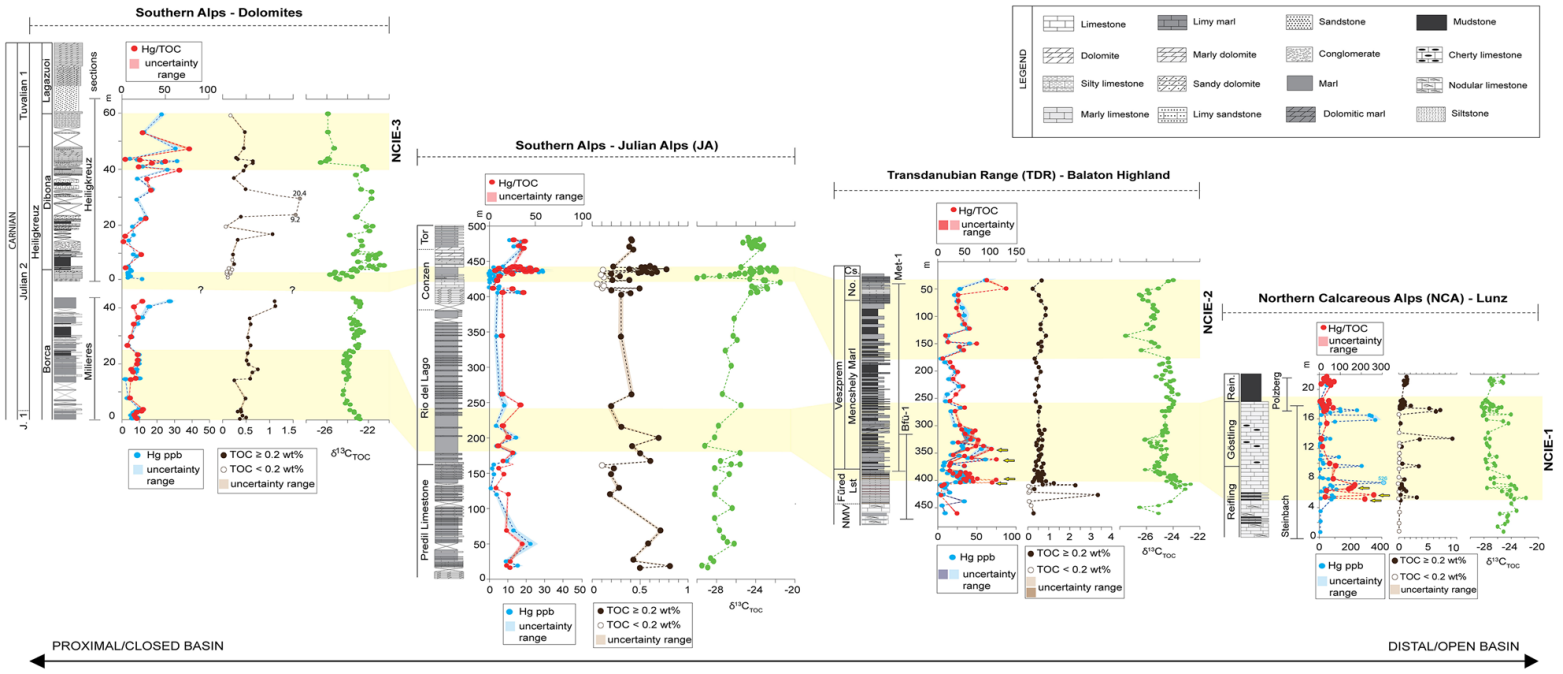
Hg, Hg/TOC, and C-isotope data from the Western Tethys. Hg has been normalized for TOC > 0.2 wt% (Fig. 2). C isotope data and stratigraphic correlations are from ref. ^6–8^. The 10% Hg concentration uncertainty is indicated on each Hg data plot in the form of shaded, semitransparent bounds; the ± 0.02 wt% uncertainty on TOC measurements is also shown by semitransparent bounds; and the uncertainty envelope for each Hg/TOC value is shown on each Hg/TOC plot by a pale red field. Note that the TOC uncertainty bounds are visible only for section Julian Alps (JA) at this scale. The yellow arrows indicate the onset of Hg/TOC peaks in the NCA and TDR before and through the facies change.

In the studied sections of the Western Tethys, the measured Hg concentrations are < 100 ppb, except those of the Northern Calcareous Alps which included the highest values (up to 526 ppb) (Figs. 2, 3). The average Hg concentration (29 ppb) in the Carnian rocks of the Western Tethys is lower than that measured in some modern sediments^33^ (average Hg = ca. 40–60 ppb) and in clastic rocks covering other Phanerozoic events linked to major volcanic activity^28^ (average Hg = 62.4 ppb), but is closer to the average Hg concentrations found in limestones across the same Phanerozoic events^28^ (average Hg = 34.3 ppb). All Hg and TOC results are in the Supplementary Tables S1–S4and Supplementary Figures S1–S4.

Low Hg concentrations are also coupled to generally low TOC values (< 1 wt%), with only 11% of all samples showing TOC above 1 wt% (Fig. 2). Hg/TOC values for TOC > 0.2 wt%, i.e., above the recommended lower limit for TOC normalization ^28^, are on average lower (38.2 ppb/wt%) than those recorded during other Phanerozoic events (average Hg/TOC = 71.9 ppb/wt%) linked to major volcanic activity, although most among them are linked to subaerial rather than submarine LIPs^28^.

In the composite section of the Dolomites, the Hg concentrations are very low (Supplementary Table S1 and Fig. S1; Fig. 3), with a maximum value of 33 ppb and an average of 9.2 ppb. Hg only shows a slight increase (from 6.5 to 27 ppb) in the upper part of the section (the upper Dibona Member of the Heiligkreuz Formation) and reaches its maximum value (33 ppb) at 40–50 m, within the NCIE-3 (Fig. 3). TOC values range from 0.1 to 20.5 wt%. Two high TOC of 9.2 wt%. and 20.4 wt% are linked to the presence of abundant wood particles, as observable in these rock samples simply by eye. In the upper part of the section (40–60 m, NCIE-3), an increase in Hg/TOC to > 20 ppb/wt% is recorded at the NCIE-3 and is parallel to the rise in Hg concentrations (Fig. 3).

The Hg concentrations of the samples from the Julian Alps are low (Supplementary Table S2 and Fig. S2; Fig. 2), with a maximum value of 27 ppb and an average of 10 ppb. TOC is also low, with values ranging from 0.2 to 0.8 wt%. Just before and at the end of the NCIE-2, Hg and Hg/TOC values show three- to sixfold increases in the samples from the Julian Alps, as Hg values rise from < 5 ppb up to 30 ppb and Hg/TOC from ca. 14 ppb/wt% up to a maximum of 49 ppb/wt% (from 435 to 440 m) (Fig. 3).

Hg values through the Balaton Highland boreholes of the Transdanubian Range show a maximum value of 61 ppb (Supplementary Table S3 and Fig. S3) with an average of 25.5 ppb (Fig. 2). A sharp Hg increase (from 5.7 to 61 ppb) is recorded in the upper Füred Limestone and the base of Mencshely Marl Member of Veszprem Formation (from 400 to 340 m) where the NCIE-1 starts (Fig. 3). Most of the TOC data are below 1 wt%. Hg/TOC shows a major increase (from 9.5 to 113 ppb/wt%) at the onset of the NCIE-1 (between 400 to 340 m; Fig. 3).

In the Lunz composite section of the Northern Calcareous Alps, the Hg concentrations vary from 2 to 526 ppb (Supplementary Table S4 and Fig. S4), with most of the data lower than 150 ppb. A sharp increase in Hg is observed from 7 ppb in the lowermost part of the Reifling Formation (at 3.5 m) to 78 ppb in the upper part of Reifling Formation (at 5 m), where the onset of the NCIE-1 is recorded (Fig. 3). Hg reaches a maximum value (526 ppb) in the uppermost Reifling Formation. At the start of the Göstling Member (onset of CPE at Julian 1–Julian 2 boundary) at 9.6 m, the third increase of Hg (from 4.3 to 274 ppb) is recorded. Throughout the Göstling Member, sporadic sharp increases of Hg (127, 358, and 240 ppb at 10.8, 15.8, and 17 m, respectively) are observed (Fig. 3). TOC content varies from 0.2 to 9.3 wt%, with most of the values below 2.5 wt%. The Hg/TOC is consistently lower than the average value (average ~ 55 ppb/wt% in Hg/TOC ratios with TOC > 0.2) with major spikes in the uppermost parts of Reifling Formation (from 5 to 10 m) coincident with the start of the NCIE-1 (Fig. 3). Notably, the very high Hg values (> 300 ppb, with a max of 526 ppb) occurring within the Göstling Member are associated with too low TOC (< 0.2 wt%) for reliable normalization (see Supplementary Fig. S4).

## Discussion

In modern and ancient sediments Hg tends to be preferentially bound to organic matter, and for this reason Hg is generally normalized to TOC content^28,33^. In the studied successions, Hg and Hg/TOC overall show similar trends and peaks (Fig. 3), suggesting that higher Hg deposition is not predominantly controlled by variations in TOC content. TOC concentrations are generally low and fall within a narrow range of values (Fig. 2), making it challenging to evaluate the exact relationships between Hg and TOC through the different sample sets.

Particular environmental and depositional conditions can enhance local drawdown of Hg and its accumulation in sediments without the need for an actual net increase of atmospheric Hg input (e.g., ref.^33^). Marine redox chemistry is a controlling factor in Hg burial. For example, the development of strong euxinic (free sulfide) conditions may lead to enhanced pyrite formation, which may facilitate Hg deposition^29,33–35^. However, the samples analysed here are all similar mudstones, thus excluding a lithological control (e.g., limestone *vs* clastic) on Hg concentration^26,36^. A small number of marl samples from the Northern Calcareous Alps have very high Hg concentrations and relatively low TOC values (Figs. 2 and 3), which could indicate the presence of Hg bound to other sulfide-mineral phases, e.g., pyrite^30^. Nevertheless, in the Northern Calcareous Alps—and also in all the other studied basins—there is evidence against (semi-)permanent euxinic conditions (which would promote Hg being hosted in sulfides^29,33^), for example, the presence of bioturbation and benthic fauna (*Halobia* and benthic foraminifera^37^) in the same strata that record the Hg spikes.

A general shift in basin-wide depositional style, from carbonate-dominated to mainly terrigenous sedimentation, is observed in the Northern Calcareous Alps and Transdanubian Range at the onset of the CPE that followed the initial C-isotope perturbations. This is related to increased continental runoff driven by an enhancement of the hydrological cycle^7^, but we note that the Hg/TOC peaks in the Northern Calcareous Alps and Transdanubian Range start before and continue through the facies change (Fig. 3). In the Transdanubian Range, this sedimentological change was also accompanied by a change in clay mineralogy, with the increase of kaolinite in the Veszprém Marls^38^. Hg can be captured by clay minerals, as observed across the Cretaceous—Palaeogene boundary and the Toarcian OAE^26^. However, in the Carnian sequences, the Hg (and Hg/TOC) starts to rise before the change in clay mineralogy, and published Al concentrations measured on the same samples from MET-1 core^22^ show no relation to Hg (Supplementary Figure S3). Hence, although part of the Hg could be hosted in other, still unidentified minerals, the absence of euxinic facies and changes in clay mineralogy directly correlated to Hg variations suggest that in the studied basins Hg is preferentially hosted in organic matter, as in the majority of modern and ancient sediments^28,33^.

Differences in position, Hg concentrations and magnitude of the Hg/TOC spikes are observed between basins. In the Northern Calcareous Alps and the Transdanubian Range, a substantial rise of Hg/TOC is recorded at the onset of the NCIE-1, while in the Southern Alps a Hg/TOC increase is not apparent in the same stratigraphic interval (Fig. 3). Subsequent Hg/TOC increases occur during NCIE-2 in the Transdanubian Range and the Julian Alps, and during NCIE-3 in the Dolomites (Fig. 3). Collectively, this suggests that in the western Tethys Hg/TOC increased during the early stages of NCIE-1 in the Northern Calcareous Alps and Transdanubian Range, just before and during the late stage of NCIE-2 in the Julian Alps and Transdanubian Range, and in the early stages of NCIE-3 in the Dolomites.

Different Hg and Hg/TOC signals could arise due to local differential diagenesis, as intense weathering and post-depositional oxidation of organic matter can change Hg concentrations in sediments and Hg/TOC values^30,39^. Enrichments in Hg and Hg/TOC may be controlled by early diagenetic degradation of organic matter, potentially amplifying the Hg/TOC ratios, especially in samples with very low TOC^40^. The detected Hg/TOC spikes are in samples with TOC content ≥ 0.2 wt% and parallel enrichments in absolute Hg concentrations (Fig. 3). Moreover, previous biomarker and pyrolysis analyses, clay mineralogy, colour alteration index of conodont apatite, and sporomorphs coloration indicate immature to low thermal maturity for the analysed samples in the Southern Alps, Transdanubian Range, and Northern Calcareous Alps^6,8,19,38^. Therefore, we find it unlikely that degradation of organic matter was responsible for the Hg/TOC signal, and we conclude that the observed anomalies in both Hg and Hg/TOC across different basins, likely record actual changes in Hg input into the depositional environment, and not local changes in Hg drawdown and/or diagenesis.

The terrestrial Hg reservoir can modulate global marine Hg deposition^30,31^. As one of the main reservoirs of the exogenic Hg cycle, soil erosion and oxidation of terrestrial organic matter following extensive collapse of vegetation on land can result in an increase of Hg input into the marine depositional environments^33^. However, there is no evidence for such modulation during the CPE. The Carnian marks the formation of thick productive coal measures after a global “coal gap” that started at the Permian–Triassic Mass Extinction^41^, and witnessed the appearance and spread of many new land plant groups^9^ and the extensive development of soils in the Western Tethys^42,43^, suggesting an overall expansion of flora rather than a collapse.

An enhanced hydrological cycle during the CPE resulted in the expansion of large riverine systems in Pangea^9,44,45^ and consequent increases of terrigenous inputs in many basins, where these spikes of continental runoff are the distinctive signature of the CPE. The transport of terrestrial material into the basins could increase Hg concentrations in marine sediments. However, higher Hg concentrations and Hg/TOC are recorded just before, at the onset, and during the NCIEs, which all precede siliciclastic inputs^7^. As discussed above, in the Northern Calcareous Alps (Lunz) and in the Transdanubian Range (Balatonfüred) Hg and Hg/TOC start to increase at the onset of the NCIE, before the δ^13^C_TOC_ reaches its minimum values, while the subsequent increases occur during NCIE-2 in the Transdanubian Range, just before and at the end of NCIE-2 in the Julian Alps as well as early stages of NCIE-3 in the Dolomites. This implies that the episodic enhancement of continental runoff was not responsible for the initial, pre-NCIEs increases of Hg loading into the basins. However, the increased siliciclastic input that followed the initial Hg and NCIE onset, could have resulted in the transport of additional Hg in later stages of the NCIEs intervals (e.g., increases of Hg at the end of the NCIE-2 in the Julian Alps).

**Figure 4:**
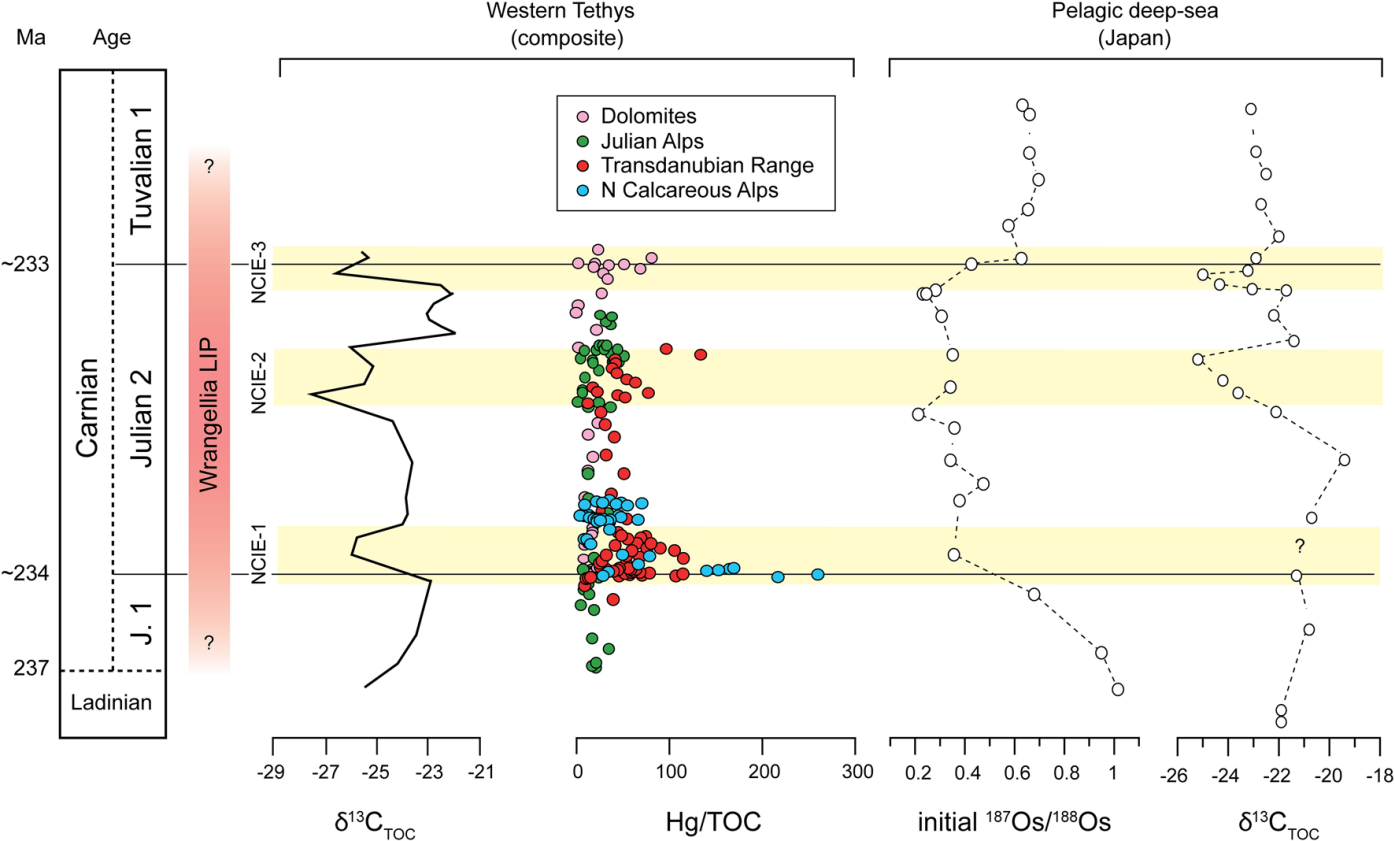
A comparison of our Western Tethys composite Hg/TOC stratigraphy with Os-isotopes through the same period associated with the Carnian Pluvial Episode recorded in Japan. Os and C-isotope data from Panthalassa are from ref.^16^. C-isotope data from the Western Tethys are from ref.^8^. The approximate period of the Wrangellia LIP emplacement is taken from refs.^6,8,9,14,16,25,48^ .

A huge amount of Hg was plausibly released by the Carnian Wrangellia oceanic plateau at the time of the CPE. Given the temporal overlap between Wrangellia LIP and the CPE, substantiated by biostratigraphic, radioisotopic, and geochemical data^9,16,25,46^, and in the absence of clear evidence for other dominant Hg drawdown and transport mechanisms, it is plausible to assume a volcanic origin of the additional Hg during the CPE. Wrangellia erupted a minimum of 1 × 10^6^ km^3^ of basalts onto the Panthalassa ocean floor (Fig. 1) at a latitude of about 10–15°N^16,18^. The remnants of Wrangellia now outcrop in northwestern America, but coeval oceanic intraplate basalts are also present in east Asia (Sambosan and Taukha belts), which could have been part of the same LIP^16^. Os isotope data from pelagic successions of Japan show a sharp ^187^Os/^188^Os increase in the Julian (Fig. 4), that is interpreted as unradiogenic Os input from Wrangellia emplacement^16^.

As with other similar oceanic plateaus^26,36^, dispersal of Hg during the initial submarine phase of Wrangellia was likely predominantly marine. Current evidence suggests that Hg records across events coeval to the emplacement of LIPs with submarine or mixed submarine/subaerial activity, show less consistent Hg perturbations than those across events coeval with subaerial continental LIPs (e.g., the Siberian Traps), i.e., with atmospheric Hg dispersal^26^. The amplified Hg signal associated with continental flood basalts may in part result from volcanic intrusions into sedimentary sequences, with coals and evaporites^47^. This potentially liberates a large quantity of previously bound Hg through melting and combustion^48^, adding to the simpler magmatic degassing thought to dominate Hg release from oceanic igneous plateaus. Moreover, the oceanic residence time of Hg is shorter (< 1000 years) than the mixing time, whilst in the atmosphere Hg in the vapor phase has a relatively longer residence time (0.5–2 years) compared to the mixing time and atmospheric Hg can thus be potentially dispersed globally in contrast to more localised dispersion for oceanic Hg^49^. As a result, Hg emitted from submarine volcanism is expected to be less efficiently and uniformly dispersed than atmospheric Hg^26,36^. Indeed, the magnitudes of the Hg/TOC peaks at events coeval with submarine LIPs (e.g., OAE 1a, OAE 1b, OAE 2) tend to be smaller (up to 200 ppb/wt%)^26,28,36,50,51^ compared to those associated with continental explosive LIPs (e.g., up to 600 ppb/wt% for the Siberian Traps)^29^. These lower magnitudes are comparable to the Hg/TOC peaks found in the Carnian (Figs. 3, 4). Hence, differential Hg and Hg/TOC signals in the different studied basins of the Western Tethys might be related to Wrangellia volcanic style, resulting in a less efficient distribution of Hg in different depositional settings. It might be hypothesized that the semi-restricted basins of the Southern Alps limited direct Hg marine influx to these basins, in contrast to the more open marine settings of the Northern Calcareous Alps and Transdanubian Range (Fig. 1). It is known that the Wrangellia switched between submarine and subaerial activity^9^. The switch in eruptive style to subaerial^52^ has not been dated yet^9^, but may have resulted in a wider atmospheric Hg dispersal, and anomalous Hg/TOC in a wider variety of continental to marine environments^26^.

**Figure 5:**
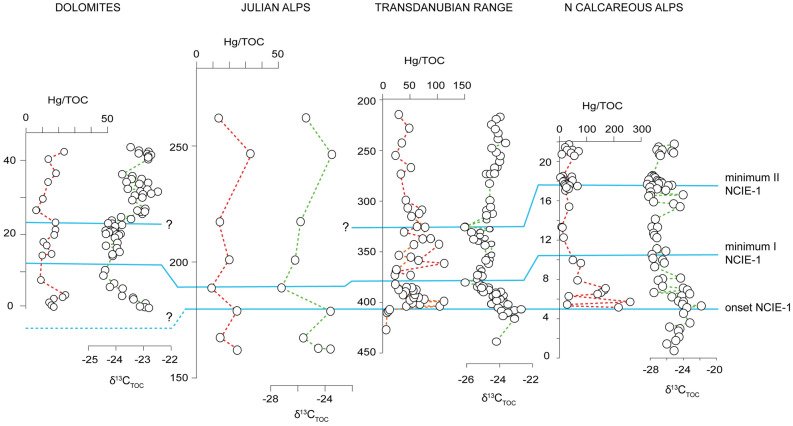
Detail of Hg/TOC and δ^13^C_TOC_ data across the NCIE-1 in the studied basins of the Western Tethys. The Hg/TOC spike associated with the NCIE-1 of the CPE could be missing in the Southern Alps (Dolomites and Julian Alps) because of the incompleteness of the succession and lower sampling resolution.

Alternatively, the lack of a Hg enrichments at the NCIE-1 in the Dolomites and Julian Alps could be explained by stratigraphic features and sampling resolution (Fig. 5). At Milieres-Dibona section (Dolomites), the onset of the NCIE-1 could be missing as the sediments below the isotope anomaly do not outcrop in the area (see ref.^7^). This is due to the fact that the uppermost San Cassiano—lowermost Heiligkreuz basinal succession of the Dolomites is represented by fine-grained shales that are easily eroded and covered by vegetation. We therefore cannot exclude the possibility that the covered sediments at the base of the section might have prevented sampling of the first Hg spike, and the onset of NCIE-1^7^. In the Julian Alps, the temporal and stratigraphic sampling resolution across NCIE-1 is limited because the sections are extremely expanded, almost vertical and poorly accessible. Here, the NCIE-1 is captured by only 4 samples, and so only these few samples were measured to assess Hg across NCIE-1, out of a total of ~ 70 m of the succession. As observed in the Transdanubian Range and Northern Calcareous Alps, Hg/TOC are not constant within the body of the NCIE-1, and the short-lived spikes may have been missed in the Julian Alps (Fig. 5).

## Conclusions

Our new data show pulses of increased Hg loading in the Western Tethys during the early Late Triassic CPE. Although muted compared to other examples in the geological record, these pulses occur in correspondence to NCIEs and we note that the Hg/TOC record is different in different basins. A rise of Hg/TOC is recorded in the more open, and complete basin successions of the Northern Calcareous Alps (Austria) and the Transdanubian Range (Hungary) at the onset of the first NCIE of the CPE, but the same spike is not recorded during the same interval in the more restricted basins of the Southern Alps (Italy). This geographical distribution might be an artifact of incomplete sections or low temporal sampling resolution. However, it is also consistent with a primary signal as major interbasinal differences in Hg concentrations and Hg/TOC are recorded during other Mesozoic LIP events linked to emplacements of oceanic plateaux, and are explained by the relatively inefficient dispersal of Hg from submarine volcanism^26,27,53^. Overall, our data is consistent with the hypothesis that pulses of Wrangellia volcanic activity triggered multiple injections of isotopically light C into the atmosphere–ocean system that led to associated environmental perturbations.

## Methods

### Hg analysis

A total of 243 samples were analysed for Hg concentrations using a Lumex RA-915 Portable Mercury Analyser with PYRO-915 Pyrolyzer at the University of Oxford, using the method described by ref. ^54^. Samples were powdered with an agate mortar, then an aliquot of 50–250 mg was weighed into a glass boat before being placed into the pyrolyzer and heated to 700 °C. Volatilized elementary Hg was quantified via atomic absorption spectrometry. At the start of each run and throughout the measurement sequences (every 10 samples), paint-contaminated soil (NIST 2587; 290 ppb Hg) standards were analysed, using masses ranging from 10 to 90 mg, to calibrate the Lumex. The analysed standards indicate reproducibility was generally better than 10% for Hg concentrations. TOC normalization of Hg was applied when TOC was above or equal to 0.2% following the approach recommended by ref.^28^. For the Transdanubian Range Hg/Al has been calculated using published elemental data of ref.^22^, which were measured on the same core material. The Hg data are coupled to the organic carbon δ^13^C data previously generated on the same samples^7,8^.

### Analysis of total organic carbon (TOC)

An Elementar Soli TOC Cube was used to determine Total Organic Carbon (TOC) in rock samples at the University of Ferrara. The analyser is equipped with two combustion units: a dynamic heater able to raise the temperature from ambient to 900 °C and a post-combustion zone kept at a constant temperature of 800 °C, containing a platinum catalyst to achieve complete oxidation of all combustion products released by the dynamic heater. Combustion takes place in pure oxygen at a flow rate of 150 ml/min. An infrared detector detects the formed CO_2_. About 500 mg of each sample were weighed into stainless steel crucibles that were heated prior to analysis to avoid contamination by C residues. Values are reported as the mean of duplicate analysis. The sample weight depended on the C content and could be extended up to 1 g in cases where the C contents were low (equal or below 0.1 wt%). A standard of calcium carbonate (CaCO_3_, Calciumcarbonat, Elementar) and a soil standard (CaCO_3_, Bodenstandard, Elementar with approximately 1.3% C content) were analysed prior, between, and after each run. Temperature-dependent differentiation of total carbon (DIN19539) has been applied for determining the total organic carbon content as described by ref.^55^. The dynamic temperature ramping method starts at about 100 °C. After introducing the sample, the temperature is increased at a rate of 90 °C/min up to 600 °C for the determination of TOC. The average standard deviation (SD), based on replicate analyses of nine samples and a soil standard (Bodenstandard), was ± 0.02 wt%.

## Supplementary Information

Supplementary Information.
