# Supplementary material for: Mercury deposition in Western Tethys during the Carnian Pluvial Episode (Late Triassic)
Source: Sci Rep. 2021 Aug 30;11:17339. doi: 10.1038/s41598-021-96890-8 (PMC8405686; doi:10.1038/s41598-021-96890-8)
Supplement: Supplementary file 1 — Supplementary Information. [file 41598_2021_96890_MOESM1_ESM.pdf]

SUPPLEMENTARY INFORMATION

Mercury deposition in Western Tethys during the Carnian Pluvial Episode (Late Triassic)

Mina Mazaheri-Johari[1]*, Piero Gianolla[1], Tamsin A. Mather[2], Joost Frieling[2], Daoliang Chu[3], and Jacopo Dal Corso[3]*

[1]Department of Physics and Earth Sciences, University of Ferrara, Ferrara, Italy.

[2]Department of Earth Sciences, University of Oxford, South Parks Road, Oxford, UK

[3]State Key Laboratory of Biogeology and Environmental Geology, School of Earth Sciences, China University of Geosciences, Wuhan, China.

*emails: mzhmni@unife.it; j.dalcorso@cug.edu.cn

EXTENDED GEOLOGICAL SETTING

1. Southern Alps (Italy)

1.1. Dolomites

The Dolomites region, located in the northcentral part of Southern Alps (Bosellini et al., 2003), displays an impressive and outstanding exposure of Triassic rocks of the Alps. During the Middle–Late Triassic, the area occupied a northern equatorial position in the western margin of the Tethys Ocean (Fig. 1a) (Muttoni et al., 2015). In the Early Carnian (Julian), the paleotopography was characterized by an emerged land (Adriatic foreland) towards the present South, then attached and isolated rimmed microbial carbonate platforms (Cassian Dolomite) and relatively deep basins, characterized by mixed carbonate-siliciclastic sedimentation (San Cassiano Formation) (Masetti et al., 1991; De Zanche et al., 1993; Gianolla et al., 1998a; Keim et al., 2006; Stefani et al., 2010; Mietto et al., 2012). Near the end of the Early Carnian, a consistent siliciclastic input to marginal basins associated with the general demise of high relief carbonate platforms (Keim et al., 2001; Dal Corso et al., 2012; Gattolin et al., 2015) caused the infilling of the intraplatform basins by the Heiligkreuz Formation and the flattening of Early Carnian topography (Pisa et al., 1980; De Zanche et al., 1993; Gianolla et al., 1998a,b, 2018; Preto and Hinnov, 2003; Keim et al., 2006; Neri et al., 2007; Breda et al., 2009; Stefani et al., 2010; Dal Corso et al., 2015, 2018; Gattolin et al., 2015). The Heiligkreuz Formation is thus characterized by a complex lithostratigraphic architecture with different members recording mainly the interplay between climatic forcing

and relative sea level oscillations (Preto and Hinnov, 2003; Neri et al., 2007; Breda et al., 2009; Stefani et al., 2010; Gattolin et al., 2015; Gianolla et al., 2018). The Borca Member documents the onset of local anoxia, a change from microbial to skeletal carbonate factory, and the basin infilling phase by skeletal carbonates and/or siliciclastics. The Dibona Member documents an overall transgressive phase and is marked by terrigenous pulses in the lower and uppermost parts. The last members of the unit are the Lagazuoi Member and the Falzarego Sandstone, documenting a regressive phase with progradation of mixed carbonate-terrigenous shallow water depositional systems.

Above the Heiligkreuz Formation, continental to shallow-marine siliciclastic-carbonates of Travenanzes Formation (Tuvalian) were deposited in a marginal marine dryland coastal system (Breda and Preto, 2011). Several studies such as those carried out by Breda et al. (2009), Dal Corso et al. (2018), and Maron et al. (2017) performed a high-resolution biostratigraphic framework of this succession in the Dolomites area. The base of Heiligkreuz Formation (Borca member) is assigned to the Julian 1 - 2 boundary (*T. aonoides*/*A. austriacum* ammonoid zones) while the upper part (upper Dibona Member and Falzarego/Lagazuoi members) belongs to the Tuvalian 1 (*T. dilleri* ammonoid zone) (Fig. 1b). In this study we have analysed two stratigraphic sections that together form a composite section of the entire Heiligkreuz Formation.

The Milieres section (Dal Corso et al., 2012, 2018), placed just below the Dibona section (De Zanche et al, 1993; Gianolla et al., 1998a; Preto and Hinnov, 2003; Neri et al., 2007; Breda et al., 2009; Gattolin et al., 2015), is a succession of marls and limestones deposited in a relatively deep basin, recording the boundary between San Cassian Formation and the lowermost part of the Borca Member of Heiligkreuz Formation, the age is within the *A. austriacum* ammonoid zone (Julian 2) (see discussion in Dal Corso et al., 2018). The Heiligkreuz section (Dal Corso et al., 2018), also known as Peraguda section (Koken, 1913), Santa Croce/Heiligkreuz section (Gianolla et al., 1998b) or Kreuzkofel section (Keim et al., 2001, 2006) encompasses the uppermost Borca, Dibona, and Lagazuoi members of the Heiligkreuz Formation, the age is within the *A. austriacum* ammonoid zone (Julian 2) for the Borca and part of the Dibona members while the uppermost Dibona and Lagazuoi members belong to the *T. dilleri* ammonoid zone (Tuvalian 1) (Gianolla et al., 1998b; De Zanche et al., 2000; Dal Corso et al., 2018).

A total number of fifty samples analysed in this study come from this composite section: 20 samples come from the Milieres section and 30 samples come from the Heiligkreuz section.

1.2. Julian Alps

The studied sequence in the Julian Alps is located in the Cave del Predil area (formerly Raibl), near Tarvisio, where the historical type-area of the Carnian stage has been defined

(Mojsisovics, 1869; Wörhmann, 1894; Assereto et al., 1968; Lieberman, 1980). In this area, a well-preserved carbonate platform-basin depositional system records microbial platform demise, basin infilling by siliciclastics, transition to mixed terrigenous-skeletal carbonate ramp system, and finally microbial carbonate platform recovery (Schulz, 1970; De Zanche et al., 2000; Gianolla et al., 2003; Caggiati et al., 2018; Dal Corso et al., 2018).

The carbonate platform (Schlern Dolomite or “Dolomia Metallifera” Auctorum) is a high-relief microbial buildup and is interfingered the Predil Limestone, a dysoxic-anoxic unit made up of laminated limestones, marly limestones, marls, and thin to coarse calcarenites. The latter unit is sharply overlain by the Rio del Lago Formation, which consists at the base by a silty interval (“Barren beds” Auctorum), then by subtidal marly shales, with marly and calcareous intercalations in the upper part. The unit is lapping on the slope of the demise carbonate platform (De Zanche et al., 2000). According to Dal Corso et al. (2018), the Rio del Lago Formation represents the onset of CPE by the infilling of a relatively deep basin, which had almost been completed by the deposition of overlying Conzen Formation (De Zanche et al., 2000). Above, a deepening upward succession of marls, siltstone, and marly limestone, the Tor Formation, comprises the Julian – Tuvalian boundary (Lieberman, 1980; De Zanche et al., 2000) and the rest of Tuvalian is represented by the Portella Dolomite, the conformably overlying basinal Carnitza Formation (*Subbullatus* to *Anatropites* Ammonoid zones), and the interfingered Dolomia Principale carbonate platform (De Zanche et al., 2000; Gianolla et al., 2003; Caggiati et al., 2018) (Fig. 1b).

In the Cave del Predil area, the Rio Conzen and neighboring Rio delle Cascate sections (De Zanche et al., 2000; Roghi, 2004) have been sampled. The sections are very close to each other and a good correlation between them can be achieved with marker beds. They encompass the Predile Dolomite (Julian 1 = *Trachyceras* zone), the Rio del Lago, the Conzen, and part of the Tor formations (Julian 2 = *A. austriacum* zone). Fifty-eight samples analysed in this study come from this composite section which was previously studied for stratigraphy, palynology and carbon-isotope records by De Zanche et al. (2000), Roghi (2004), and Dal Corso et al. (2018).

2. Transdanubian Range (Hungary)

The Transdanubian Range (TDR) is a NE-SW trending chain of moderately elevated mountains located in the north-western part of Hungary, developed in a length of about 200 km. It is made up predominantly of Middle and Late Triassic shallow-marine carbonates and coeval relatively deep basinal sediments representing a large segment of the Neotethys passive margin (Haas et al., 1995; Gawlick, 2000; Mandl, 2000; Haas et al., 2012). During Early Carnian a significant change in the lithofacies occurred in the Balaton Highland in the TDR as pelagic limestones (Füred Limestone) passes into a thick marl succession (Veszprém

Marl Formation (VMF)) with a marked siliciclastic input (Fig. 1b) (Budai and Haas, 1997; Rostási et al., 2011; Dal Corso et al., 2018; Baranyi et al., 2019). This major change in the sedimentation is associated with the CPE climatic shift from arid to more humid conditions as documented by clay mineralogy (Rostási et al., 2011) and by palynomorph assemblages (Baranyi et al., 2019). In the Balaton Highland, the VMF with a variety of thickness (from 100 to 600 m) is comprised of four members: Mencshely Marl Member, Nosztor Limestone Member, Buhimvolgy Breccia Member and Csicso Marl Member (Góczán et al., 1991, Góczán and Oravecz-Scheffer, 1996a,b; Budai et al., 1999; Haas and Budai, 1999). The basal part of the Veszprém Marl (Mencshely Marl Member), included of a relatively thick marl-dominated succession with some intercalations of sandstone, is separated from the pelagic marls of the upper part (Csicso Marl Member) by a 20 m-thick pelagic limestone member (Nosztor Limestone) (Góczán et al., 1991; Budai et al., 1999). Above, the Sàndhoregy Formation (Góczán and Oravecs-Scheffer,1996; Nagy, 1999) is a succession of shallow-water shales and carbonates, which represent the final stage of the infilling of the intraplatform basins during late Carnian (Tuvalian). On top of this unit the Main Dolomite deposited from late Tuvalian to the Norian (Budai and Haas, 1997) (Fig. 1b). The Carnian successions of the TDR are also biostratigraphically well constrained with ammonoids, sporomorphs, and conodonts (Budai et al., 1999; Dal Corso et al., 2018; Baranyi et al., 2019). A total number of seventy-three samples analyed in this study come from two core materials (Rostási et al., 2011). 34 samples come from the Met-1 core which encompasses the Veszprém Marl Formation and 39 samples are from the Bfü-1 borehole included of Füred Limestones and the base of Mencshely Marl Member of Veszprém Marl Formation.

3. Northern Calcareous Alps (Austria)

The Lunz area, located in the eastern Northern Calcareous Alps, is well-known for containing coal seams with numerous remnants of Late Triassic plant fossils and reptiles (Verloop, 1908; Tollmann, 1976; Dobruskina, 1998; Pott et al., 2008). The Early Carnian stratigraphy of the Lunz nappe (Austria), represents a succession of carbonate–siliciclastic sedimentary rocks from deep water to delta and carbonate shelf basins (Rüffer and Bechstädt, 1998; Roghi et al., 2010; Dal Corso et al., 2015; Mueller et al., 2016). The Upper Ladinian–Lower Julian sequence is characterized by deep-water nodular limestones of the Reifling Formation (greyish-coloured filament wackestones), which is overlain by the Göstling Member (Mueller et al., 2016). The laminated dark mudstones and grainstones of the Göstling Member deposited in a deep and low-energy setting represent the demise of microbial platform and the temporary replacement of a skeletal carbonate factory (Mueller et al., 2016), nutrient excess, oxygen depletion in the basin that mark the onset of CPE (Hornung and Brandner, 2005; Hornung et al., 2007a,b; Dal Corso et al., 2015). The sequence continues with the

Reingraben Formation, overlain by the coarse terrigenous Lunz Formation. The deep-marine deltaic sandstones and siltstones of Lunz Formation with a turbiditic origin change to deltaic sediments through basin fill and the infilling process of basins was completed by the shale and coal member of the Lunz Formation (Köppen, 1997; Hornung and Brandner, 2005; Wessely, 2006; Pott et al., 2008; Roghi et al., 2010; Mueller et al., 2016). Above, the succession continues with Opponitz Formation and Hauptdolomit (Tollmann, 1976, Roghi et al., 2010) (Fig. 1b). The age of the studied succession is assigned to Carnian using ammonoid and sporomorph biostratigraphy (Roghi et al., 2010; Dal Corso et al., 2015; Mueller et al., 2016). Sixty-two samples analysed in this study come from two stratigraphic sections in this area: Steinbach section and Polzberg section. The Steinbach section encompasses the Reifling Fm. and part of the Göstling Mb. (Julian 1 = *Trachyceras* zone, Julian 2 = *A. austriacum* zone) while in the Polzberg section the uppermost part of the Göstling Mb. and the lower part of the Reingraben Fm. are exposed (Julian 2 = *A. austriacum* zone).

4. Location of the studied sections and cores

Milieres section, Dolomites, Italy: 46°31’41.60″N, 12°4′7.34″E

Heiligkreuz section, Dolomites, Italy: 46°35′50.56″N, 11°56′46.22″E

Rio Conzen section, Cave del Predil, Julian Alps, Italy: 46°26′38.43″N; 11°56′46.22″E

Rio delle Cascate section, Cave del Predil, Julian Alps, Italy: 46°26′10.9″N; 13°35′3.54″E

Steinbach section (= Göstling section in Dal Corso et al., 2015), Northern Calcareous Alps, Austria: 47°48′22.6″N, 14°57′3.44″E

Polzberg section, Northern Calcareous Alps, Austria: 47°53′4.09″N; 15°4′28.17″E

BFÜ-1 and MET-2 cores are stored at Szépvízér (repository of the Hungarian Office for Mining and Geology) in Hungary.

For more information on how to find and reach the studied sections, please contact P. Gianolla (piero.gianolla@unife.it) or J. Dal Corso (j.dalcorso@cug.edu.cn).

SUPPLEMENTARY TABLES (Hg is ppb, TOC in wt%)

Table S1 Hg, TOC, and Hg/TOC data from the Dolomites (Milieres and Heiligkreuz sections), Southern Alps, Italy.

SAMPLE	Meter	Hg ppb	TOC wt%
**MILIERES**			
MI 20/1	0.22	6.8	0.40
MI 20/2	0.62	9.3	0.56
MI 20/3	1.52	6.4	0.45
MI 20/4	2.62	7.1	0.43
MI 20/4B	2.9	7.8	0.35
MI 20/5	3.56	10.7	0.44
MI 20/6	7.6	4.4	0.51
MI 20/7	14.3	2.9	0.29
MI 20/8	14.62	10	0.64
MI 20/9	16.96	8.4	0.67
MI 20/10	17.84	8.9	0.84
MI 20/11	19.74	10	0.59
MI 20/12	21.12	10	0.55
MI 20/13	23.12	10.3	0.58
MI 20/14	26.44	3.9	0.65
MI 20/15	29.34	5.9	0.58
MI 20/16	34.02	9	0.65
MI 20/17	36.3	12	0.63
MI 20/17B	40.2	15.7	1.18
MI 20/18	42.2	27.7	1.17
**HEILIGKREUZ**			
SSC 1	0.4	0.2	
SSC 2	0.92	0.9	0.11
SSC 3	1.32	2.5	0.11
SSC 4	1.9	0.5	0.08
SSC 6	2.45	0.9	0.11
SSC 7A	2.65	0.4	0.18
SSC 9	3.29	3	0.18
SSC 10	3.73	2	0.11
SSC 14	4.9	0.9	0.21
SSC 22	8.86	5.7	
SSC 26	9.77	5.3	0.22
SSC 30	14.2	0.6	0.32
SSC 31	16.24	4.4	1.02
SSC 31C	19.24	3.6	0.07

SSC 31 F	22.53	11	0.39
SSC 32	22.93	13	9.30
SSC 34	29.32	6.7	20.47
SSC 39	32.59	17	0.50
SSC 42	39.72	27	0.40
SSC 45	42.22	12	0.64
SSC 46	42.77	33	0.64
SSC 47	43.34	6.7	0.30
SSC 48	43.6	1.2	0.27
SSC 49	47.31	32	0.41
SSC 50	53.1	11	0.45
SSC 52	59.66	23	0.14

Table S2. Hg, TOC, and Hg/TOC data from the Julian Alps (Cave del Predil), Italy.

Sample	Meter	Hg ppb	TOC wt%
PRSC2	16	11	0.52
PRSC3	18.6	15	0.82
PRSC4	24.8	9	0.41
PRSC7	49.7	21	0.61
PRSC11	68.7	13	0.73
PRSC14	119.8	4.9	0.25
PRSC15	128.4	1.8	0.31
PRSC16	148.3	3.3	0.21
PRSC17	156.8	2.6	0.29
PRSC18A	162	3	0.12
RLSR29	166.8	9.5	0.64
RLSR30	178.3	13	0.53
RLSR31	188.4	4.3	0.47
RLSR32	200.4	14	0.71
RLSR34	217	4.2	0.31
RLSR35	246	8.3	0.25
RLSR36	261.8	6.1	0.46
RLSR40	344.3	4.3	0.31
LG1Ca	405.5	5	0.36
LG1Cb	405.5	17	0.47
LG5	411.2	5.4	0.23
LG6	411.9	1.8	0.53

LG7	413.3	0	0.14
LG8	414.4	6	0.14
LG10	420	0.8	0.11
LG11	422.8	2.9	0.41
LG12	423.2	2	0.29
LG13	424.8	2.1	
LG23	428.3	1.8	0.19
LG15	428.3	3.6	0.35
LG24	429	0.3	
LG26	431.5	0.4	
LG27	431.7	6.8	0.26
LG37	432.6	0	0.10
LG28	432.8	1.9	
LG30	434.6	8.2	0.45
LG17	435.2	26	0.70
LG31A	435.3	19	0.48
LG31B	435.3	13	0.71
LG38	435.3	27	0.61
LG32	436.1	4.1	0.60
LG39	436.2	4.2	0.16
LG40	436.7	0.8	
LG41	437.2	27	0.55
LG42	438.4	24	0.69
LG18	439.4	3.9	0.41
LG43	439.6	17	0.81
LG19	439.8	16	0.53
LG45	440.1	16	0.55
LG20	440.2	22	0.51
LG46	440.3	20	0.62
LG21	441.5	2.6	
LG49	441.7	7.4	0.29
LG52	468.2	17	0.48
LG58	471.3	13	0.42
LG66	478.5	17	0.46
LG68	479.9	11	0.45

Table S3. Hg, TOC, and Hg/TOC from the TDR (Balaton Highland) boreholes (Bfü-1, Met-1), Hungary.

SAMPLE	Meter	Hg ppb	TOC wt%
**BFU-1**			
SZBF 2	6	35	0.80
BFU 1	10.5	34	0.67
BFU 2	16	38	0.50
BFU 4	22.5	31	0.43
BFU 6	27.4	33	0.60
SZBF 22	31.2	17	0.65
BFU 7	32.5	28	0.56
BFU 9	36.5	8.9	0.39
BFU 11	40.3	7.9	0.40
BFU 12	45.2	6.3	0.32
BFU 13	46.5	29	0.71
SZBF 29	47	24	0.47
BFU 14	50.5	12	0.46
BFU 15	51.5	31	0.52
BFU 16	52.5	39	0.74
SZBF 35	54	22	0.57
BFU 17	56.5	20	0.57
BFU 18	57.5	34	0.60
BFU 19	59.2	25	0.46
SZBF 41	59.5	32	0.50
BFU 20	61	28	0.38
SZBF-45	62	32	0.68
BFU 21	63	38	0.34
BFU 22	65	29	0.57
BFU 23	66	43	0.64
SZBF 53	67.8	35	0.87
SZBF 54	68.5	23	0.22
BFU 24	71.5	12	1.25
SZBF 56	73.2	5.7	0.71
SZBF 57	74.7	11	2.27
SZBF 59	76.8	6.5	0.03
SZBF 61	79.5	9.5	0.13
SZBF 68	85.3	14	0.07
SZBF 71	88	1.3	0.03
SZBF 77	93	6.8	3.36
SZBF 85	105.2	34	0.06
SZBF 88	112.5	5.6	0.18
SZBF 96	126.5	9.5	0.28

MET-1			
Met-1 1	33.8	61	0.65
Met-1 4	49.4	28	0.21
Met-1 7	61	22	0.56
Met-1 10	72	25	0.62
Met-1 13	85	31	0.85
Met-1 16	98	34	0.83
Met-1 19	114.5	32	0.61
Met-1 21	122.9	38	0.62
Met-1 22	135.1	13	0.89
Met-1 23	147.03	13	0.66
Met-1 24	150	41	0.55
Met-1 25	155.8	27	0.66
Met-1 26	162	31	0.62
Met-1 27	177.4	3.2	0.34
Met-1 28	184	9.3	0.38
Met-1 31	192.9	16	0.56
Met-1 33	202	23	0.59
Met-1 36	215.1	14	0.49
Met-1 40	228.4	27	0.56
Met-1 43	242.7	12	0.34
Met-1 46	255.4	9.3	0.40
Met-1 48	267.2	29	0.56
Met- 1 51	273.5	17	0.52
Met-1 54	299.5	22	0.52
Met-1 55	309	40	0.55
Met-1 56	312.3	38	0.57
Met-1 57	325.9	44	0.70
Met-1 58	331	19	0.49
Met-1 59	337.5	59	0.67
Met-1 60	343.2	55	0.53
Met-1 61	359.5	38	0.59
Met-1 62	361.7	61	0.54
Met-1 63	370	17	0.73
Met-1 64	373.9	35	0.67

Table S4. Hg, TOC, and Hg/TOC data from the NCA (Steinbach and Polzberg sections), Austria.

SAMPLE	Meter	Hg ppb	TOC wt%
STI 2	0.71	4.5	0.04
STI 6	2.1	6.7	0.08
STI 10	3.52	7	0.11

STI 16	5.1	78	0.36
STI 17	5.25	5.9	0.13
STI 18	5.4	96	3.38
STI 19	5.67	81	0.31
STI 21	6.2	8.5	0.12
STI 22	6.25	38	1.13
STI 23	6.48	109	0.78
STI 24	6.6	33	0.22
STI 25	6.87	83	0.51
STI 27	7.05	72	0.43
STI 28	7.25	526	0.13
STI 29	7.86	59	0.89
STI 30	8	24	
STI 31	8.08	6.5	0.11
STI 33	9.24	2.3	0.08
STI 34	9.63	274	3.49
STI 35	9.95	25	0.51
STI 36	10.1	23	0.17
STI 37	10.48	4.3	0.16
STI 38	10.84	127	
STI 39	11.06	10	0.14
STI 40	12.21	4.8	0.34
STI 41	12.4	3.8	0.16
STI 42	12.67	5.6	0.11
STI 43	13.26	36	3.49
STI 43a	13.3	70	9.30
STI 46	15.35	8.2	0.23
STI 47	15.65	99	
STI 49	15.85	358	
STI 51	16.42	331	
STI 52	16.5	324	
STI 53	16.54	11	0.15
STI 54	16.85	68	
STI 55	17.03	137	6.42
STI 56	17.18	240	7.32
STI 57A	17.35	134	5.32
POZ 2	17.48	13	0.20
POZ 3	17.52	15	0.42
POZ 4	17.54	13	0.59
POZ 5	17.62	6.9	0.25
POZ 6	17.67	42	2.20
POZ 7	17.73	8.9	0.37
POZ 10	17.93	14	1.01

POZ 11	18	4.5	0.29
POZ 12	18.07	28	0.61
POZ 14	18.29	6.6	0.89
POZ 15	18.37	2	0.63
POZ 16	18.43	14	0.31
POZ 17	18.5	6.4	0.50
POZ 18	20.56	49	0.90
POZ 19	20.71	11	1.16
POZ 20	20.86	56	1.32
POZ 21	21.01	70	1.03
POZ 22	21.15	37	1.38
POZ 23	21.15	34	0.74
POZ 24	21.44	28	1.36
POZ 26	21.73	46	1.31

SUPPLEMENTARY FIGURES

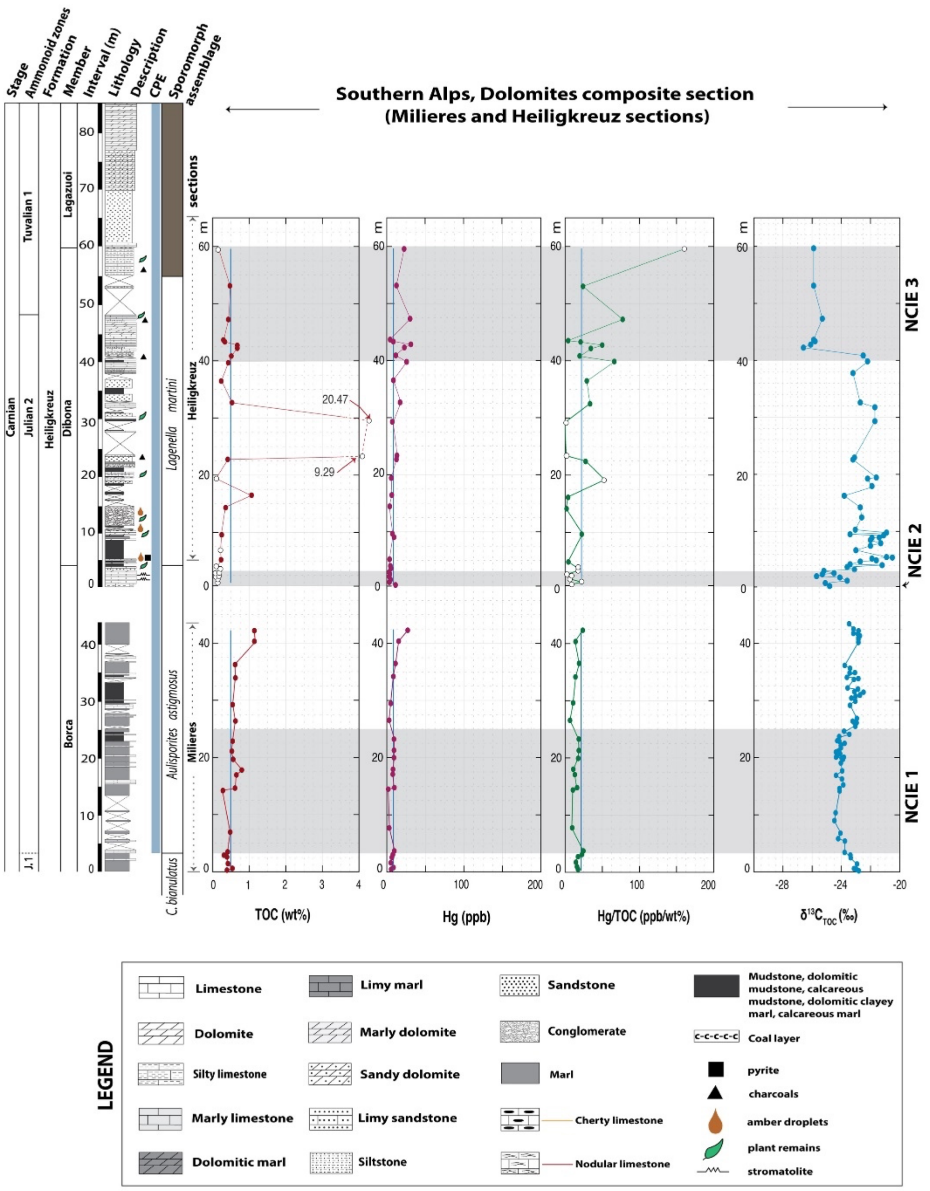

Fig. S1. Mercury geochemistry of the Southern Alps, Dolomites composie section. The TOC chemostratigraphy, Hg enrichments and Hg/TOC ratios produced in this study. Milieres section: Lithostratigraphy modified from Dal Corso et. al., (2018); Biostratigraphy and organic carbon-isotope curves from Dal Corso et al. (2012, 2018). The grey bar shows the negative carbon isotope excursion (NCIE-1) detected by Dal Corso et al. (2018). J. 1 = Julian 1 = *Trachyceras* zone, Julian 2 = *Austrotrachyceras austriacum* zone. Heiligkreuz section: Lithostratigraphy modified from Dal Corso et al., (2018). Biostratigraphy and organic carbon-isotope curves from Dal Corso et al. (2018). Grey bars show the two negative carbon isotope excursions (NCIE-2 and NCIE-3) detected by Dal Corso et al. (2018). The dark brown area in the palynostratigraphical zonations represent intervals of uncertain palynostratigraphic attribution. White circles represent TOC values below 0.2 wt% and their corresponding Hg/TOC ratios. The two outlier points in TOC (9.2 and 20.4) are from wood-rich samples. Vertical blue lines represent the average values of each dataset (only TOCs>0.2 are included in average). J-1 = Julian 1= *Trachyceras* zone, Julian 2 = *Austrotrachyceras austriacum* zone; Tuvalian 1 = *Tropites dilleri* zone.

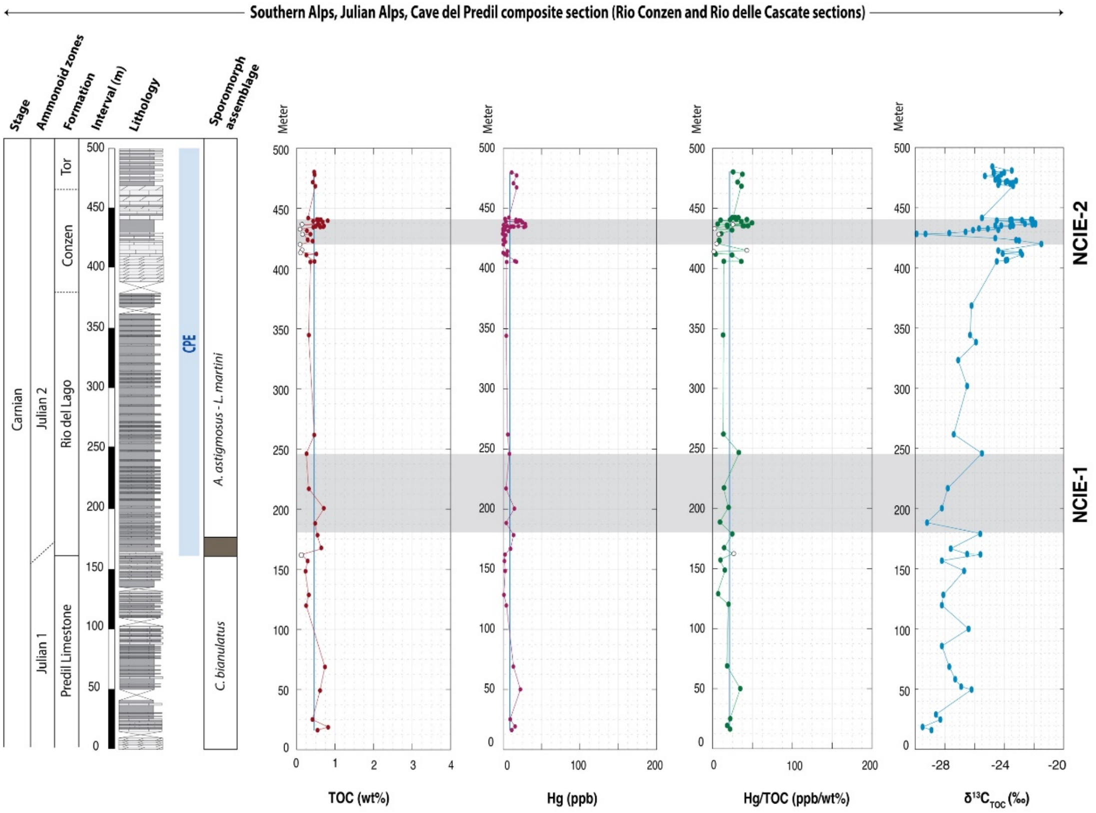

Fig. S2. Mercury geochemistry of the Julian Alps, Cave del Predil composite section (Rio Conzen and Rio delle Cascate sections). The TOC chemostratigraphy, Hg enrichments and Hg/TOC ratios produced in this study. Lithostratigraphy modified from De Zanche et al. (2000), Roghi (2004), and Dal Corso et al., (2015, 2018); Biostratigraphy and carbon-isotope curves from Dal Corso et al. (2018). Grey bars show the two negative carbon isotope excursions (NCIE) detected by Dal Corso et al. (2018). The dark brown area in the palynostratigraphical zonations represent intervals of uncertain palynostratigraphic attribution. White circles represent TOC values below 0.2 wt% and their corresponding Hg/TOC ratios. Vertical blue lines represent the average values of each dataset (only TOCs>0.2 are included in average). Julian 1 = *Trachyceras* zone, Julian 2 = *Austrotrachyceras austriacum* zone. The legend of the lithostratigraphic units is the same as in Figure S1.

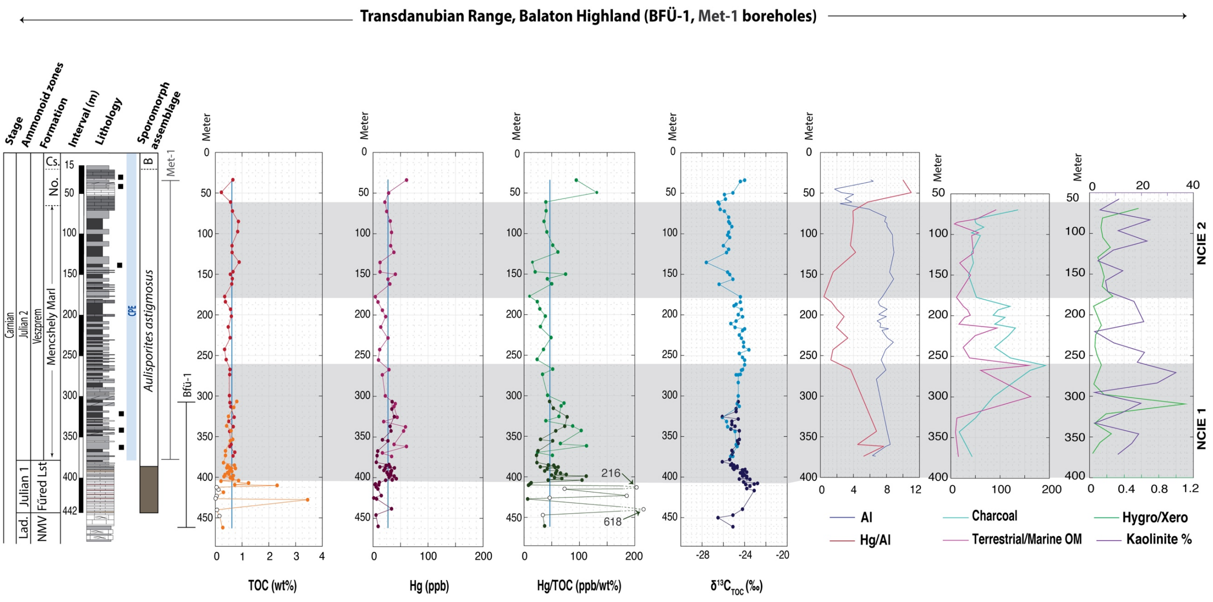

Fig. S3. Mercury geochemistry of the Transdanubian Range, Met-1 (light colours) and Balatonfüred-1 (Bfü-1, darker colours) boreholes. The TOC chemostratigraphy, Hg enrichments and Hg/TOC ratios produced in this study. Lithostratigraphy from Rostási et al. (2011), Dal Corso et al. (2018) and Baranyi et al. (2019); palynostratigraphy from Roghi et al. (2010), Dal Corso et al. (2018), Baranyi et al. (2019) and organic carbon-isotope curves from Dal Corso et al. (2015, 2018). The charcoal abundances, terrestrial/marine organic matter distribution, hygrophytic/xerophytic ratios, aluminum, and kaolinite contents are from Baranyi et al. (2019). Grey bars show the two negative carbon isotope excursions (NCIE) detected by Dal Corso et al. (2015, 2018). The dark brown area in the palynological zonation represent intervals of uncertain palynostratigraphic attribution. White circles represent TOC values below 0.2 wt% and their corresponding Hg/TOC ratios. Vertical blue lines represent the average values of two datasets (only TOCs>0.2 are included in average). Julian 1 = *Trachyceras* zone, Julian 2 = *Austrotrachyceras austriacum* zone. Abbreviations: No = Nosztor Limestone, Cs = Csicsó Marl, Hygro/Xero = Hygrophytic/Xerophytic, Al = Aluminium content, B = *Lagenella martini* palynozone, Lad. = Ladinian (*Protrachyceras* zone). The legend of the stratigraphic units is the same as in Figure S1.

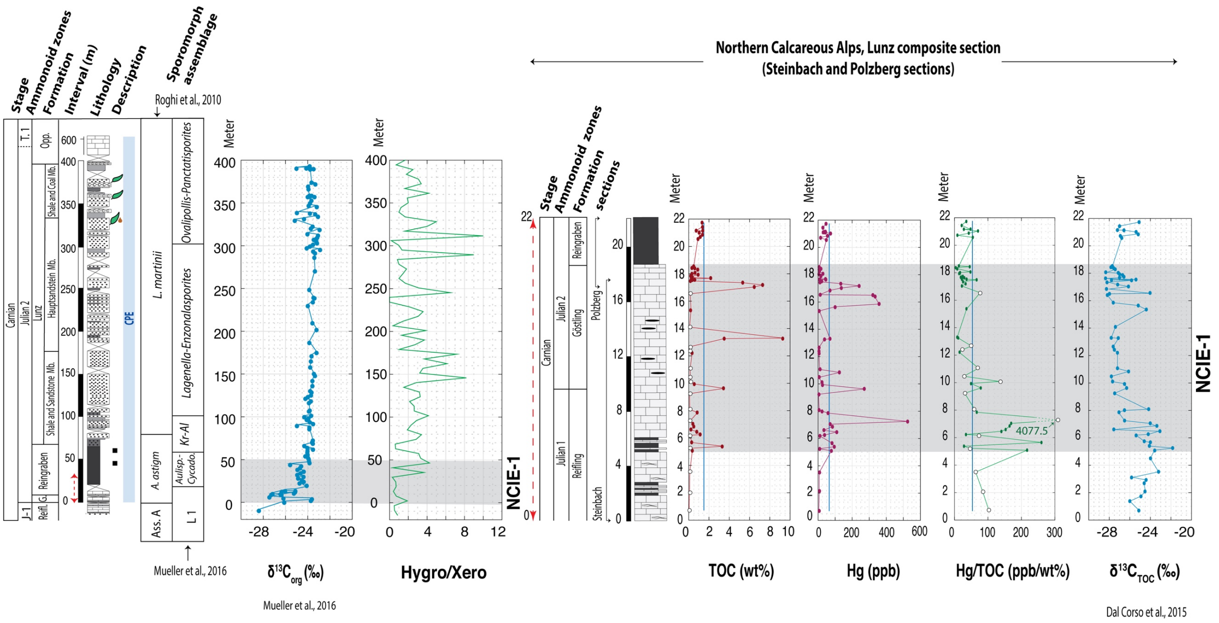

Fig. S4. Mercury geochemistry of the Northern Calcareous Alps, Lunz composite section. The TOC chemostratigraphy, Hg enrichments and Hg/TOC ratios produced in this study. Lithostratigraphy modified from Dal Corso et al. (2015) and Mueller et al. (2016); Organic carbon isotope curves from Dal Corso et al. (2015) and Mueller et al. (2016). Biostratigraphy from Roghi et al. (2010) and Mueller et al. (2016). The Hygrophytic/Xerophytic ratios from Mueller et al. (2016). Grey bars show the negative carbon isotope excursion detected by Dal Corso et al. (2015) and Mueller et al. (2016). White circles represent TOC values below 0.2 wt% and their corresponding Hg/TOC ratios. Vertical blue lines represent the average values of each dataset (only TOCs>0.2 are included in average). Abbreviations: Reifl. = Reifling Formation, G. = Göstling Member, Opp. = Opponitz Formation, J-1 = Julian-1 = *Trachyceras* zone, Julian 2 = *Austrotrachyceras austriacum* zone, T. 1: Tuvalian 1 = *Tropites dilleri* zone. The legend of the stratigraphic units is the same as in Figure S1.

SUPPLEMENTARY REFERENCES

Assereto, R., Desio, A., di Colbertaldo, D., and Passeri, L.D., 1968, Note illustrative della Carta Geologica d'Italia: Foglio 14 A Tarvisio. Servizion Geologico Italiano, Roma, p. 70.

Baranyi, V., Rostási, Á., Raucsik, B., and Kürschner, W.M., 2019b, Palynology and weathering proxies reveal climatic fluctuations during the Carnian Pluvial Episode (CPE) (Late Triassic) from marine successions in the Transdanubian Range (western Hungary): Global and Planetary Change, v. 177, p. 157-172, https://doi.org/10.1016/j.gloplacha.2019.01.018.

Bin, C., Xiaoru, W., and Lee, F.S.C., 2001, Pyrolysis coupled with atomic absorption spectrometry for the determination of mercury in Chinese medicinal materials: Analytica Chimica Acta, v. 447, p. 161-169, https://doi.org/10.1016/S0003-2670(01)01218-1.

Bosellini, A., Gianolla, P., and Stefani, M., 2003, Geology of the Dolomites: Episodes, v. 26, p. 181-185, https://doi.org/10.18814/epiiugs/2003/v26i3/005.

Breda, A., and Preto, N., 2011, Anatomy of an Upper Triassic continental to marginal-marine system: The mixed siliciclastic-carbonate Travenanzes Formation (Dolomites, Northern Italy): Sedimentology, v. 58, p. 1613–1647, https://doi.org/10.1111/j.1365-3091.2011.01227.x.

Breda, A., Roghi, G., Furin, S., Meneguolo, R., Ragazzi, E., Fedele, P., and Gianolla, P., 2009, The Carnian Pluvial Event in the Tofane area (Cortina d'Ampezzo, Dolomites, Italy): Geo.Alp, v. 6, p. 80–115.

Budai, T., and Haas, J., 1997, Triassic sequence stratigraphy of the Balaton Highland, Hungary: Acta Geologia Hungarica, v. 40, p. 307–335.

Budai, T., Császár, G., Csillag, G., Dudko, A., Koloszár, L., and Majoros, Gy, 1999, Geology of the Balaton Highland (Explanation to the Geological Map of the Balaton Highland, 1:50000): Budapest: Geological Institute of Hungary, Special Publication 197, p. 171–257.

Caggiati, M., Gianolla, P., Breda, A., Celarc, B., and Preto, N., 2018, The start-up of the Dolomia Principale/Hauptdolomit carbonate platform (Upper Triassic) in the eastern Southern Alps: Sedimentology, v. 65, p. 1097-1131, https://doi.org/10.1111/sed.12416.

Dal Corso, J., et al., 2018, Multiple negative carbon-isotope excursions during the Carnian Pluvial Episode (late Triassic): Earth-Science Reviews, v. 185, p. 732–750, https://doi.org/10.1016/j.earscirev.2018.07.004.

Dal Corso, J., Gianolla, P., Newton, R.J., Franceschi, M., Roghi, G., Caggiati, M., Raucsik, B., Budai, T., Haas, J., and Preto, N., 2015, Carbon isotope records reveal synchronicity between carbon cycle perturbation and the "Carnian Pluvial Event" in the Tethys realm (Late Triassic): Global and Planetary Change, v. 127, p. 79–90, https://doi.org/10.1016/j.gloplacha.2015.01.013.

Dal Corso, J., Mietto, P., Newton, R.J., Pancost, R.D., Preto, N., Roghi, G., and Wignall, P.B., 2012, Discovery of a major negative $\delta^{13}C$ spike in the Carnian (Late Triassic) linked to the eruption of Wrangellia flood basalts, Geology, v. 40, p. 79–82, https://doi.org/10.1130/G32473.1.

De Zanche V., Gianolla P., Mietto P., Siorpaes C., and Vail P.R., 1993, Triassic sequence stratigraphy in the Dolomites (Italy): Memorie Scienze Geologiche, v. 45, pp. 1-27.

De Zanche, V., Gianolla, P., and Roghi, G., 2000, Carnian stratigraphy in the Raibl/Cave del Predil area (Julian Alps, Italy): Ecologae Geologicae Helvetiae, v. 93, p. 331-347.

Dobruskina, I.A., 1998, Lunz flora in the Austrian Alps-a standard for Carnian floras: Palaeogeography, Palaeoclimatology, Palaeoecology, v. 143, p. 307–345, https://doi.org/10.1016/S0031-0182(98)00116-3.

Gattolin, G., Preto, N., Breda, A., Franceschi, M., Isotton, M., and Gianolla, P., 2015, Sequence stratigraphy after the demise of a high-relief carbonate platform (Carnian of the Dolomites): Sea-level and climate disentangled: Palaeogeography, Palaeoclimatology, Palaeoecology, v. 423, p. 1–17, https://doi.org/10.1016/j.palaeo.2015.01.017.

Gawlick, H. J., 2000, Paläogeographie der Ober-Trias Karbonatplattform in den Nördlichen Kalkalpen: Mitteilungen der Österreichischen Geologischen Gesellschaft, v. 44, p. 45-95.

Gianolla, P., De Zanche, V., and Mietto, P., 1998a. Triassic Sequence Stratigraphy in the Southern Alps (Northern Italy): Definition of Sequences and Basin Evolution, in: de Graciansky, P.-C, Hardenbol, J., Jacquin, T., Vail, P.R. (Eds.), Mesozoic and Cenozoic Sequence Stratigraphy of European Basins. SEPM Special Publications, pp. 719–747. https://doi.org/10.2110/pec.98.02.0719.

Gianolla, P., De Zanche, V., and Roghi, G., 2003, An Upper Tuvalian (Triassic) Platform-Basin System in the Julian Alps: the start-up of the Dolomia Principale (Southern Alps, Italy): Facies, v. 49, p. 135 – 150, https://doi.org/10.1007/s10347-003-0029-7.

Gianolla, P., Morelli, C., Cucato, M., and Siorpaes, C., 2018. Note Illustrative - Foglio 016 Dobbiaco, in: Carta Geologica d'Italia alla Scala 1:50000. ISPRA, Roma.

Gianolla, P., Ragazzi, E., and Roghi, G., 1998b, Upper Triassic amber from the Dolomites (Northern Italy). A paleoclimatic indicator?: Rivista Italiana di Paleontologia e Stratigrafia, v. 104, p. 381-390, https://doi.org/10.13130/2039-4942/5340.

Góczán, F., and Oravecz-Scheffer A., 1996a, Tuvalian Sequence of the Balaton Highland and the Zsámbér Basin. Part I: Litho-bio and chronostratigraphic subdivision: Acta Geologica Hungarica, v. 39/1, pp. 1-31.

Góczán, F., and Oravecz-Scheffer A., 1996b, Tuvalian Sequence of the Balaton Highland and the Zsámbér Basin. Part II: Characterization of sporomorph and foraminifer assemblages, biostratigraphic, palaeogeographic, and geohistoric conclusion: Acta Geologica Hungarica., v. 39/1, pp. 33-101.

Góczán, F., Oravecz-Scheffer, A., and Csillag, G., 1991, Balatoncsicsó, CsukrétiÁrok cordevolei és juli képződményeinek biosztratigráfiai jellemzése (The stratigraphic characterization of the Cordevolian and Julian formations of Csukrét ravine, Balatoncsicsó). Ann. Rep. Hung. Geol. Inst. on. 1989, pp. 241-323.

Grasby, S. E., Sanei, H., Beauchamp, B., and Chen, Z., 2013, Mercury deposition through the Permo–Triassic biotic crisis: Chemical Geology, v. 351, p. 209-216, https://doi.org/10.1016/j.chemgeo.2013.05.022.

Grasby, S.E., Beauchamp, B., Bond, D.P.G., Wignall, P.B., Talavera, C., Galloway, J.M., Piepjohn, K., Reinhardt, L., and Blomeier, D., 2015, Progressive environmental deterioration in northwest- ern Pangea leading to the latest Permian extinction: Geological Society of America Bulletin, v. 127, p. 1331–1347, https://doi.org/10.1130/ B31197.1.

Haas, J., Budai, T., and Raucsik, B., 2012, Climatic controls on sedimentary environments in the Triassic of the Transdanubian Range (Western Hungary): Palaeogeography, Palaeoclimatology, Palaeoecology, v. 353–355, p. 31–44, https://doi.org/10.1016/j.palaeo.2012.06.031.

Haas, J., Kovács, S., Krystyn, L., and Lein, R., 1995, Significance of late Permian–Triassic facies zones in terrane reconstructions in the Alpine–North Pannonian domain: Tectonophysics, v. 242, p. 19–40, https://doi.org/10.1016/0040-1951(94)00157-5.

Hornung, T., and Brandner, R., 2005, Biochronostratigraphy of the Reingraben Turnover (Hallstatt Facies Belt): local black shale events controlled by regional tectonics, climatic change and plate tectonics: Facies, v. 51, p. 460-479, https://doi.org/10.1007/s10347-005-0061-x.

Hornung, T., Brandner, R., Krystyn, L., Joachimski, M.M. and Keim, L., 2007b, Multistratigraphic constraints on the NW Tethyan "Carnian Crisis": The Global Triassic, New Mexico Museum of Natural History Bulletins, v. 4, p. 9–67.

Hornung, T., Krystyn, L., and Brandner, R., 2007a, A Tethys-wide mid-Carnian (Upper Triassic) carbonate productivity decline: evidence for the Alpine Reingraben Event from Spiti (Indian Himalaya): Journal Asian Earth Science, v. 30, p. 285–302, http://dx.doi.org/10.1016/j.jseaes.2006.10.001.

Keim, L., Brandner, R., Krystyn, L., and Mette, W., 2001, Termination of carbonate slope progradation: an example from the Carnian of the Dolomites, Northern Italy: Sedimentary Geology, v. 143, p. 303-323, https://doi.org/10.1016/S0037-0738(01)00106-3.

Keim, L., Spöti, C., and Brandner, R., 2006, The aftermath of the Carnian carbonate platform demise: a basinal perspective (Dolomites, Southern Alps): Sedimentology, v. 53, p. 361-386, https://doi.org/10.1111/j.1365-3091.2006.00768.x.

Koken, E., 1913, Kenntnis der Schichten von Heiligenkreuz (Abteil. Stüdtirol), Abhandlungen der Kaiserlich−Koniglichen Geologischen Reichsanstalt, v. 16, p. 1-44.

Köppen, A., 1997, Faziesentwicklung in der frühen Obertrias Mitteleuropas – ein sequenzstratigraphischer Vergleich [Ph.D. thesis]: Gaea Heidelbergensis, v. 2, p. 1-233.

Lieberman, H.M., 1980, The suitability of the Raibl sequence as a stratotype for the Carnian Stage and the Julian Substage of the Triassic: Newsletters on Stratigraphy, v. 9, p. 35–42, http://doi.org/10.1127/nos/9/1980/35.

Mandl, G.W., (2000), The Alpine sector of the Tethyan shelf–examples of Triassic to Jurassic sedimentation and deformation from the Northern Calcareous Alps: Mitteilungen der Österreichischen Geologischen Gesellschaft, v. 92, p. 61-77.

Maron, M., Muttoni, G., Dekkers, M. J., Mazza, M., Roghi, G., Breda, A., Krijgsman W., and Rigo, M., 2017, Contribution to the magnetostratigraphy of the Carnian: new magneto-biostratigraphic constraints from Pignola-2 and Dibona marine sections, Italy: Newsletters on Stratigraphy, v. 50, p. 187-203, https://doi.org/10.1127/nos/2017/0291.

Masetti, D., Neri, C., and Bosellini, A., 1991, Deep-water asymmetric cycles and progradation of carbonate platforms governed by high-frequency eustatic oscillations (Triassic of the Dolomites, Italy): Geology, v. 19, p. 336-339, https://doi.org/10.1130/0091-7613(1991)019<0336:DWACAP>2.3.CO;2.

Mietto, P., et al., 2012, The global boundary stratotype section and point (GSSP) of the Carnian stage (Late Triassic) at Prati di Stuores/Stuores Wiesen section (Southern Alps, NE Italy): Episodes, v. 35, 414-430, https://doi.org/10.18814/epiiugs/2012/v35i3/003.

Mojsisovics, E.M., von, 1869, Über die Gliederung der oberen Triasbildungen der östlichen Alpen: Jahrbuch der Geologischen Bundesanstalt, v. 19, p. 91-150.

Mueller, S., Krystyn, L., and Kürschner, W.M., 2016, Climate variability during the Carnian Pluvial Phase-a quantitative palynological study of the Carnian sedimentary succession at Lunz am See, Northern Calcareous Alps, Austria: Palaeogeography, Palaeoclimatology, Palaeoecology, v. 441, p. 198–211, https://doi.org/10.1016/j.palaeo.2015.06.008.

Muttoni, G., Tartarotti, P., Chiari, M., Marieni, C., Rodelli, D., Dallanave, E., and Kirscher, U., 2015, Paleolatitudes of Late Triassic radiolarian cherts from Argolis, Greece: Insights on the paleogeography of the western Tethys: Palaeogeography, Palaeoclimatology, Palaeoecology, v. 417, p. 476-490, https://doi.org/10.1016/j.palaeo.2014.10.010.

Nagy, Z.R.,1999, Platform-basin transition and depositional models for the Upper Triassic (Carnian) Sándorhegy Limestone, Balaton Highland, Hungary: Acta Geologica Hungarica, v. 42/3, p. 267-299.

Natali, C., Bianchini, G., and Carlino, P., 2020, Thermal stability of soil carbon pools: Inferences on soil nature and evolution: Thermochimica Acta, v. 683, 178478, https://doi.org/10.1016/j.tca.2019.178478.

Neri C., Gianolla P., Furlanis S., Caputo R., and Bosellini A., 2007 - Note illustrative della Carta Geologica d'Italia. Foglio Cortina d'Ampezzo 029. Scala 1:50.000. Servizio Geologico d'Italia, 200 pp.

Pisa, G., Marinelli, M., and Viel, G., 1980, Infraraibl Group: a proposal (Southern Calcareous Alps, Italy): Rivista Italiana di Paleontologia e Stratigrafia, v. 85, p. 983-1002.

Pott, C., Krings, M., and Kerp, H., 2008, The Carnian (Late Triassic) flora from Lunz in Lower Austria: Paleoecological considerations: Palaeoworld, v. 17, p. 172-182, https://doi.org/10.1016/j.palwor.2008.03.001.

Preto, N., and Hinnov, L.A., 2003, Unraveling the origin of carbonate platform cyclothems in the Upper Triassic Dürrenstein Formation (Dolomites, Italy): Journal of Sedimentary Research, v. 73, p. 774-789, https://doi.org/10.1306/030503730774.

Roghi, G., 2004, Palynological investigations in the Carnian of Cave del Predil area (once Raibl, Julian Alps): Review of Palaeobotany and Palynology, v. 132, p. 1–35, https://doi.org/10.1016/j.revpalbo.2004.03.001.

Roghi, G., Gianolla, P., Minarelli, L., Pilati, C., and Preto, N., 2010, Palynological correlation of Carnian humid pulses throughout western Tethys: Palaeogeography, Palaeoclimatology, Palaeoecology, v. 290, p. 89–106, https://doi.org/10.1016/j.palaeo.2009.11.006.

Rostási, Á., Raucsik, B., and Varga, A., 2011, Palaeoenvironmental controls on the clay mineralogy of Carnian sections from the Transdanubian Range (Hungary): Palaeogeography, Palaeoclimatology, Palaeoecology, v. 300, p. 101–112. https://doi.org/10.1016/j.palaeo.2010.12.013.

Rüffer, T., and Bechstädt, T., 1998, Triassic sequence stratigraphy in the western part of the Northern Calcareous Alps (Austria): in: de Graciansky, P.-C, Hardenbol, J., Jacquin, T., Vail, P.R. (Eds.), Mesozoic and Cenozoic Sequence Stratigraphy of European Basins. SEPM Special Publications, v. 60, p., https://doi.org/10.2110/pec.98.02.0751.

Schulz, O., 1970, Vergleichende petrographische Untersuchungen an Karnischen Sedimenten der Julischen Alpen, Gailtaler Alpen und des Karwendels: Verh. Geol. B.-A., v. 1970, p. 165-229.

Stefani, M., Furin, S., and Gianolla, P., 2010, The changing climate framework and deposi- tional dynamics of the Triassic carbonate platforms from the Dolomites: Palaeogeography, Palaeoclimatology, Palaeoecology, v. 290, p. 43–57, https://doi.org/10.1016/j.palaeo.2010.02.018.

Tollmann, A., 1976, Analyse des klassischen nordalpinen Mesozoikums: Stratigraphie, Fauna und Fazies der Nördlichen Kalkalpen XV: Deuticke Wien, 580 p., Springer, Berlin, Heidelberg New York.

Verloop, J.H., 1908, Profil der Lunzer Schichten in der Umgebung von Lunz: Zeitschrift deutscher geologischer Gesellschaft, Monatsberichte, v. 60, p. 81-89.

Wessely, G., 2006, Niederösterreich-Geologie der Österreichischen Bundesländer: Vienna, Austria, Verlag der Geologische Bundesanstalt, 416 p.

Wörhmann, S.F. von, 1894. Die Raibler Schichten nebst kritischer Zusammenstellung ihrer Fauna: Jahrbuch der Kaiserlich-Koniglichen Geologischen Reichsanstalt, v. 43 (1893), p. 617-768.
